# A review on urban agriculture: technology, socio-economy, and policy

**DOI:** 10.1016/j.heliyon.2022.e11583

**Published:** 2022-11-12

**Authors:** Grace Ning Yuan, Gian Powell B. Marquez, Haoran Deng, Anastasiia Iu, Melisa Fabella, Reginald B. Salonga, Fitrio Ashardiono, Joyce A. Cartagena

**Affiliations:** a College of International Relations, Ritsumeikan University, Kita-ku, Kyoto 603-8577 Japan; b College of Global Liberal Arts, Ritsumeikan University, Ibaraki, Osaka 567-8570 Japan; c Graduate School of Economics, Ritsumeikan University, Kusatsu, Shiga, 525-8577 Japan; d Institute for Advanced Education and Research, Nagoya City University, Mizuho-cho, Mizuho-ku, Nagoya 467-8501 Japan; e College of Policy Science, Ritsumeikan University, Ibaraki, Osaka 567-8570 Japan; f Graduate School of Bioagricultural Sciences, Nagoya University, Furo-cho, Chikusa-ku, Nagoya 464 -8601 Japan

**Keywords:** Urban agriculture, Vertical farming, Sustainable city, Policy, Genetically modified plants

## Abstract

It has been a challenge to support the expansion of urban agriculture (UA) in cities due to its poor economic profitability. However, it is also hard to deny the increasing benefits of UA in improving the socio-environmental dimension of cities. Hence, in this review, different aspects of UA were examined to highlight its value beyond profitability such as social, health and well-being, disaster risk reduction, and environmental perspectives. A case study and relevant policies were analyzed to determine how policy makers can bridge the gap between current and future UA practices and sustainable development. Bridging these policy gaps can help the UA sector to sustainably grow and become successfully integrated in cities. Moreover, advancements in UA technologies and plant biotechnology were presented as potential solutions in increasing the future profitability of commercial UA. Consequently, as new UA-related technologies evolve, the multidisciplinary nature of UA and its changing identity from agriculture to digital technology, similarly require adaptive policies. These policies should maximize the potential of UA in contributing to resiliency and sustainability and incentivize the organic integration of UA in cities, while equally serving social justice.

## 1 Introduction

Agriculture has long been the major source of food for mankind. It has the potential to end world hunger and boost the economies of developing nations. It is also an essential industry that will remain at the center of human activity for many centuries to come. However, the agricultural system practiced today can hardly be called sustainable due to the increasing strain it puts on our planet's scarce resources. Especially with the growing population projected to peak at nearly 11 billion by 2100, agriculture will struggle to meet the needs of the world population (UN, 2019). To shoulder the increasing pressure, expansion of agriculture is necessary, but it is a challenging endeavor in the context of climate change, which requires transition to a model compatible with sustainable development. Urban agriculture (UA), which was practiced since ancient times, captured attention as a potential solution. According to Smit et al. (1996), UA can be defined as “*an industry that produces, process and markets food and fuel, largely in response to the daily demand of consumers within a town, city or metropolis, on land and water dispersed throughout the urban and peri-urban area, applying intensive production methods, using and reusing natural resources and urban wastes, to yield a diversity of crops and livestock*.” UA is being positioned as a sustainable alternative for the traditional methods that require colossal amounts of scarce natural resources such as water.

Preliminary to addressing the contemporary interests in UA, it should be noted that though not as readily exemplified in the modern, developed world, agriculture does in fact have a longstanding history in urban spaces (Smit et al., 1996). Take for instance the widespread implementation of “war gardens” in the United States during the World Wars to bolster domestic food production during times of financial hardship. These gardens were later associated with themes of victory due to their contributions to the war effort and representation of civilian patriotism (Mares and Peña, 2010). Yet another documented example is the use of garden areas in Japan during the Edo era both in and around castles which were cultivated by the local farmers and tenants. Many Japanese cities during this time had integrated land use layouts, employing a combination of farmland and residential space (Yokohari et al., 2010). As Fuji et al. (2002 in Yokohari et al., 2010) stated, such systems supplied residents not only with fresh local produce, but with improved standards of sanitation due to their simultaneous utilization of night soil as fertilizer.

When placed in modern discourses however, UA has evolved as an effective tool and commonly cited solution to many contemporary challenges. The Sustainable Development Goals (SDGs) set out by the United Nations for the year 2030 is a direct manifestation of the types of initiatives in which UA can be employed for developed and developing countries alike. Nicholls et al. (2020) examined urban and peri-urban agriculture by applying relevant sustainable development goals as a framework to consider the “synergies and tradeoffs across multiple objectives.” In doing so, the impacts of UA within society were identified in relation to specific targets such as no poverty, zero hunger, sustainable communities and cities, and climate action. As this review will seek to detail, UA's extensive relation with such a set of goals is demonstrative of the sector's significance in a sustainable future.

The growing number of urban farm initiatives may be attributed largely to its importance in food security efforts. This has coincided with a simultaneous emergence of local food production movements in developed countries with populated metropolitans (Nicholls, 2020). That is, many cities in the Global North have become isolated from the food supply chain which reduced access to commercial fresh produce, and simultaneously limited volume and variety of nutritional foods for the wider public (Opitz et al., 2016). As nearly 68% of the world's population is projected to migrate to urban areas by the year 2050, UA offers the potential to help these vulnerable, populated cities grapple with the subsequent challenge on food insecurity (Nicholls et al., 2020).

Alongside food security, and as this review will also seek to explore, urban and community farming efforts encompass a wider range of beneficial services which require appropriate implementation. In many cities, community farms have offered alternative social benefits to the residents. For example, a study conducted on farms in New York found a wide range of shared goals exhibited by the local farmers. Significantly, it outlined the numerous ways in which practitioners contributed to social, political, economic, and environmental problems external to food production. Some such activities included educational programs and workshops on health and nutrition, environmental restoration, and political activism within the realm of UA (Cohen and Reynolds, 2014).

On the contrary, when coupled with the UA's recent resurgence, the ramification of such diverse approaches and experiences has been the UA's incompatibility with a rather narrowly defined legislative system. This has in turn slowed down or completely inhibited the incorporation of initiatives into cities (Orsini et al., 2020). On the extreme end of this, farming and gardening activities can, and have, become engulfed by unchanging systems making them a part of socially unjust phenomena such as gentrification. For instance, some lower income neighborhoods in San Francisco have become subject to “environmental gentrification” on account of community garden startups which were originally intended to serve the residents. Respective municipalities began noticing the pleasant environment generated by the greenery and open space, and initiated remodeling efforts to conform the neighborhood with middle- and upper-class tastes (Marche, 2015).

Even in instances where UA is not actively contributing to gentrification processes, farms have run into other problems. For instance, small communities or family farms often utilize labor-intensive methods because of a lack of access to necessary equipment or limited awareness of more efficient alternatives. Economic viability is thus compromised due to the low efficiency of material and labor inputs (McDougall et al., 2019). Conversely, some large-scale commercial farms employ newly developed agricultural methods or advanced technological systems to manage large scale urban farms. However, many such operations are still in the developing stage, and may lack policy regulation. Because these systems are still being researched and developed, they can have unintended consequences or implications that require further alterations to make them sustainable. Barbosa et al. (2015) determined the substantial energy consumption of urban hydroponic farms as an example, which offsets its potential for greater yields and water conservation methods. Carolan (2020) also emphasized how tech-based or digital farming is often capital-intensive in nature, which lacks economic viability in the absence of guaranteed long-term profits.

This paper has conducted an integrative review of the literature to identify the multifarious aspects of UA and how these have directly or indirectly contributed to the viability of its application. It seeks to update and contribute to the UA topic by employing a multi-perspective approach and providing an integrated look into UA as a whole (Artmann and Sartison, 2018; Haigh and Amaratunga, 2010; Snyder, 2019; Torraco, 2016). To achieve this, the paper has drawn on two broad, yet related categories of literature. First, it accounts for research examining the most recent developments in the field by offering a detailed analysis of emerging practices such as vertical farming and plant biotechnology (Kalantari et al., 2018; Kwon et al., 2020; Lobato-Gómez et al., 2021; O'Sullivan et al., 2020). Having constructed a stable, conceptual framework of the most up-to-date practices, the paper turns to literature exploring the multidimensional contributions made by UA through practical applications (Carolan, 2020; Chang and Morel, 2018; Dubbeling et al., 2019; Foodtank, 2017; McClintock, 2016; McDougall et al., 2019; Siegner et al., 2018; Tomkins et al., 2019; Yoshida and Yagi, 2021). The literature is disaggregated into five major subcategories covering, economic, social, disaster risk reduction, health and wellbeing, and environmental perspectives. In building upon these observations, the final section presents possible recommendations by identifying suitable technologies and government policies that might help farmers make UA more economically viable and socially relevant moving forward.

The paper adopts a holistic approach by considering both theoretical and empirical research, with each perspective offering alternative insights into the potentials and perils of UA implementation. It therefore aims to provide an overview and analysis of relevant literature that is available to date. To this extent, the recommendations are based on and limited to the conclusions drawn by selected literature. Further empirical research would thus be required to substantiate these claims and better assess the practicality of implementation.

## 2 Recent status of urban agriculture

UA is considered a common feature of cities in developing countries. Particularly in the Global North, a resurgence of UA in recent years have been associated with socioeconomic benefits including but not limited to food security, social justice, environmental quality, and health, and in some cases “*experimenting with radical alternatives to the capitalist neoliberal organization of urban life*” (Tornaghi, 2014). Furthermore, problems associated with traditional agricultural practices, which can be separated roughly into two categories: those (1) concerning loss of wildlife to expand the arable land and (2) consequences from the intensified land use (Lubowski et al., 2006), had pushed UA as a way to lower the reliance on traditional agriculture. This interest in UA as a sustainable alternative to traditional agriculture, particularly in highly urbanized developed nations, was further highlighted due to UA's role as food source in cities where food supply had been cut due to production and logistic disruption brought by COVID-19 pandemic in 2019. Yet, while having positive prospects, UA also has its own limitations and disadvantages. First and foremost, the concern is the amount of available land in the urban area given the expansion of the cities (FAO, 2011). While the search for a solution for this problem is in progress with new technologies allowing for vertical cultivation of crops, the price of the initial setup remains a relevant concern as it will be inaccessible for the poorer population. Certain special knowledge is required for the large-scale operation of UA installations for commercial gain as well.

By utilizing innovative methods and technologies, UA can alleviate the pressure from rural agriculture and secure food supply within a sustainable framework. With industrial-scale production, rural agriculture is focusing on monocultures which sacrifices diversity of the cultivated crops and accelerates soil degradation. UA, on the other hand, can provide sufficient variety of crops and vegetables for a person's daily consumption while occupying only 10% of urban space (Hernandez and Manu, 2018).

Previously, cities were regarded as incompatible with agriculture due to the lack of available land required for farming. This perception began to change as people discovered ways to creatively use limited space, such as designing rooftop gardens and farms and adopting for agricultural practices underutilized land in the urban areas which is not sufficient for construction or other purposes. Technological advancement significantly contributed to the expansion of UA with various vertical farming techniques being developed, allowing for better management of space. Also, biotechnological advancement has been simultaneously developing and contributing to the development of more varieties of crops which can grow suitably in urban setting and conditions. Hence, this review will present advances in vertical farming and plant biotechnology which are important drivers in UA's adoption in cities.

### 2.1 Vertical farming

Vertical farming is a UA technique that allows for an indoor cultivation of crops where factors such as lighting, temperature, and nutrients can be controlled and administered with precision. This revolutionary method reduces the required amount of freshwater in addition to conservation of land and soil. The technology is constantly being improved, and as a result urban farmers can choose from different types of vertical farming techniques varying in their levels of sophistication and cost. Thus, even without specific allocation of land by the municipal governments, UA farmers can still integrate sustainable agricultural practices into cities and engage in commercial activities. Given that UA expansion will continue, vertical farming can become a reliable source of food for urban dwellers.

**Figure 1:**
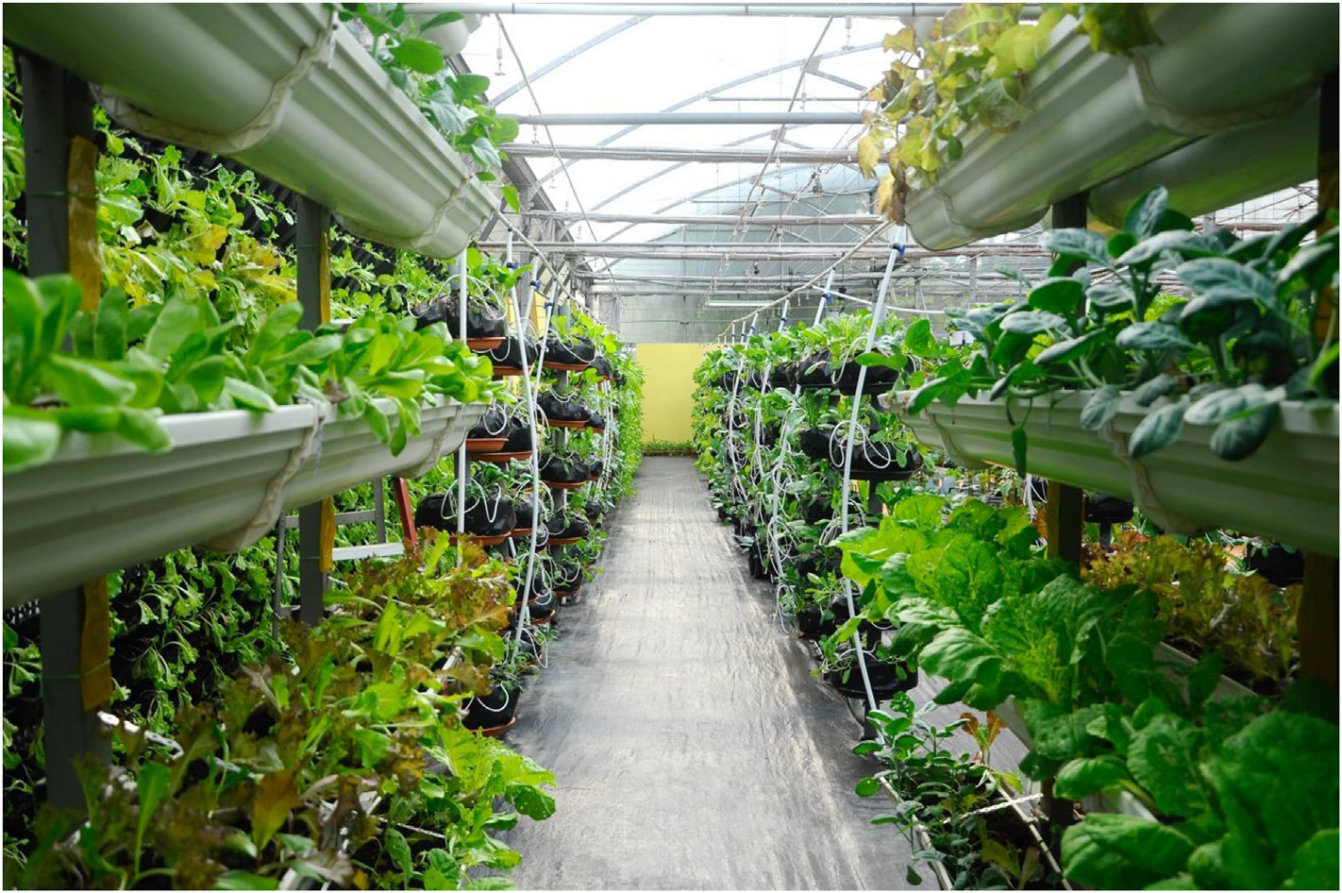
A vertical farm of vegetable crop to increase food resiliency of cities. Photo by Aisyaqilumaranas/Shutterstock.com.

In the conditions of the urban space where land is an expensive asset, urban farmers who pursue commercial gains inevitably encounter the problem of finding locations large enough to ensure profit for the business. Technologies of vertical farming present a viable solution which also has a potential to offer a sustainable solution for the future development of agriculture. Traditional horizontal spread of the farming fields over the centuries caused great damage to the environment, encroaching on the forest territories thus destroying and upsetting other ecosystems (WHO, 2005). Vertical farms, on the other hand, do not require large horizontal space and are able to fit in the urban landscapes thus potentially eliminating the need for further sprawl of the traditional rural farms (Figure 1). However, it is not the only benefit vertical farms have to offer to environmental sustainability. It also allows to sufficiently reduce the amount of the freshwater consumption while still producing greater yields as compared to conventional farming methods (Kalantari et al., 2017, 2018). One of the relevant concerns regarding discussion of vertical farming, and urban farming in general, is their economic competitiveness vis-a-vis conventional rural farming that can produce a larger amount of yield due to the vast space the farmlands occupy. However, it is argued that urban farms can achieve economic sustainability even without additional sources of income if they undergo a process of farm diversification (Yoshida et al., 2019).

Among other advantages of vertical farming is that the food is free from harmful pesticides and herbicides since in the controlled conditions of indoor farming, the risk of pest infection is substantially reduced, which maximizes the overall nutrition of the product (Al-Kodmany, 2018). However, pests such as downy mildew, molds, spider mites, insects, and others, have still been reported to occur and their control follows the same chemical pesticides as employed in conventional farms. But the controlled environment of vertical farms made it easier for the use of biological pest control as an environmentally benign option (Currey, 2017), which can be integrated in the system using banker plants (Roberts et al., 2020). Nevertheless, for commercial-scale vertical farms, it is still more economical and environmentally safe to employ prevention strategies against pests than combatting them (Currey, 2017). Also, the use of fertilizers in vertical farming has different forms with each method having its own benefits and limits. In this review, hydroponics, aeroponics, aquaponics, and digeponics will be on focus.

#### 2.1.1 Hydroponics

**Figure 2:**
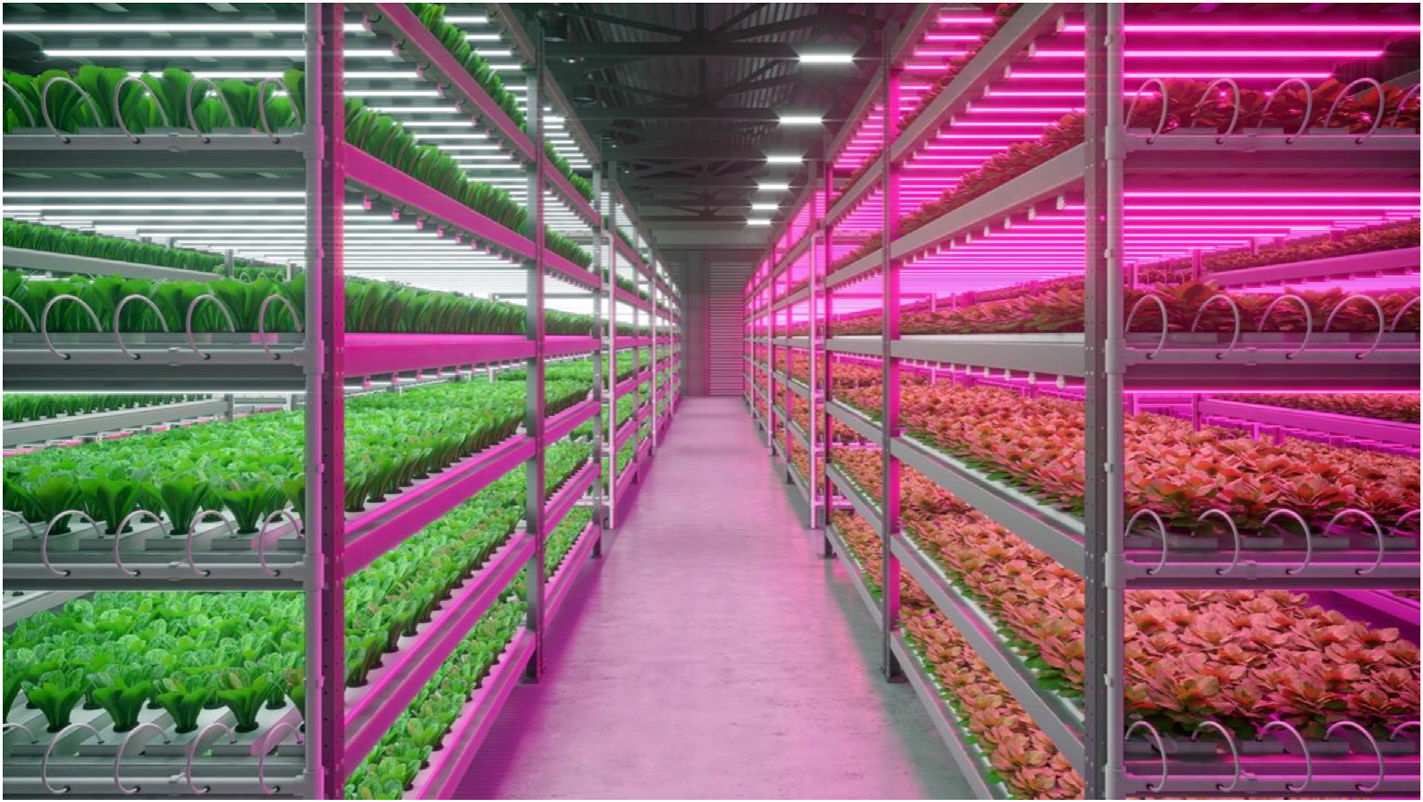
A hydroponic farm of leafy vegetables using LED light. Photo by Nikolay_E/Shutterstock.com.

Hydroponics can be considered a form of vertical farming that grows plants in nutrient solutions instead of soil, which can be done with or without the use of inert medium. This is a relatively easy technique that eliminates the possibility of soil-borne disease and stimulates faster growth of the plants (Figure 2). However, while it reduces the amount of water required for irrigation and prevents pests from infecting the plants, it does not rule out the possibility of water-borne diseases, which might spread quicker than soil-borne and destroy the entire yield (Sharma et al., 2018).

Moreover, hydroponics offers farmers a wide variety of other production advantages that should also be noted briefly here. The most prominent of which is its efficient allocation of land and water. This is notable when compared with conventional farming methods that are often land-use intensive and may utilize inefficient means of irrigation (Barbosa et al., 2015). Additionally, many studies have highlighted significantly higher output rates for hydroponic farms. For example, Barbosa et al. (2015) found that lettuce yields from hydroponic farms were 11 times higher than traditional methods. However, this came at the cost of higher energy consumption. According to the same study, yields of lettuce per greenhouse unit can have energy demand up to 90,000 ± 11,000 kJ/kg/y while traditional methods only demand up to 1100 ± 75 kJ/kg/y. This translates to 82 times more energy consumption of hydroponic farms compared to the traditional ones. On the side note, researchers and scientists are continuously developing optimization schemes for efficient energy consumptions. One example of this scheme is the use of Internet of Things (IoT) based systems which can also provide solutions towards agricultural modernization, as cited in Khudoyberdiev et al. (2020). These are in the form of sensors and microcontrollers, which can be found in smart cities, environmental monitoring, smart farming, and are responsible for improving the overall system efficiency and automization processes (Mehmood et al., 2019). However, the integration of IoT may further diminish the environmental performance of hydroponic systems when energy sources are of non-renewable. But if these are replaced with renewable alternatives, GHG emissions and the negative environmental impact of hydroponic farms can be greatly reduced (Martin et al., 2019). The same observation on the analysis of overall efficiency of urban hydroponics was pointed out by Romeo et al. (2018). They echoed notions of higher energy demands of hydroponic farms. But, since the system is powered by electricity which can easily be generated by renewable sources, the hydroponic system can perform better than the heated greenhouses and open field farms in terms of higher production yield and minimal environmental impact (Romeo et al., 2018). The higher production yield of 23 crops in hydroponic system compared with soil-based farming has been further summarized by Sathyanarayana et al. (2022).

#### 2.1.2 Aeroponics

Aeroponics is another form of vertical farming that does not require soil but, unlike hydroponics, uses mist sprayed on the roots of the plants to supply necessary nutrients. This method requires even less water than hydroponics and 95 % less than traditional agricultural methods which makes it a viable solution in cities experiencing water scarcity (Al-Kodmany, 2018, Figure 3). A study of Otazu (2010) shows that in aeroponic systems, only 1/10^th^ to 1/30^th^ of water are used in field production of crop plants such as potatoes. The thin layer of water acts as a buffer to the plants and allows oxygenation to the roots. In addition, on top of eliminating the soil-borne diseases, it also solves the problem of water-borne diseases which is still a possibility with the hydroponic method.

**Figure 3:**
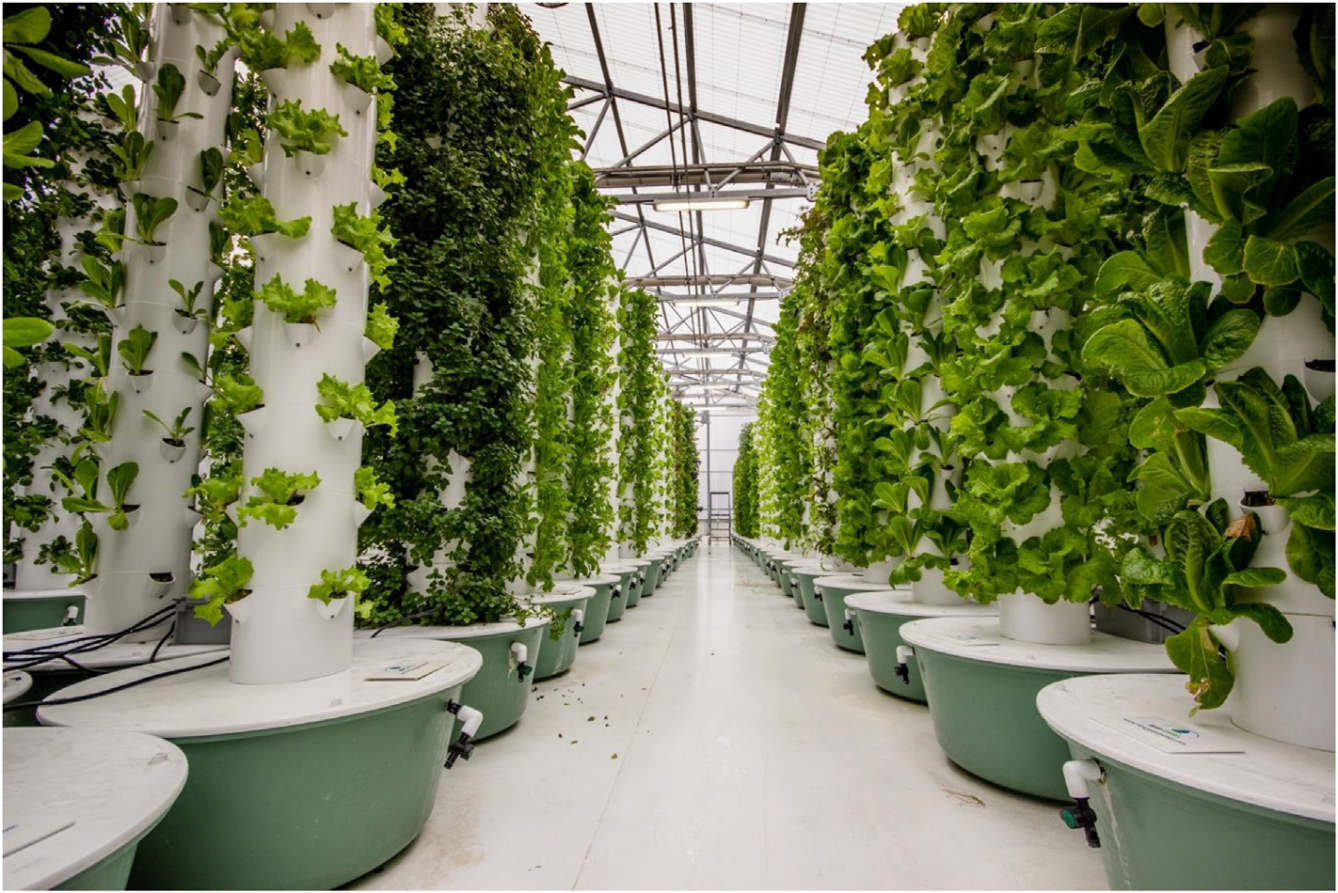
An aeroponic farm of leafy vegetables where water is directly sprayed to the roots. Photo by Globe Guide Media Inc/Shutterstock.com.

In Thailand, Srihajong et al. (2006) established a mathematical model for operating an aeroponic system for agricultural products. In their simulation, total electric energy consumption per day is 8.46kWh, with an initial cost for heat pipes of 13 000 Baht (40 Baht ≅ 1 USD). When compared with hydroponic system, the start-up cost of aeroponics is more expensive. Aeroponic systems also require constant monitoring, particularly when pumps used in aeroponic systems operate under a steady high pressure (80 pounds per square inch) with required nutrient flow. The high pressure is required to spray an ideal droplet size (20–100 microns) of water and nutrient mixtures for plant growth (Gopinath et al., 2017). The droplet size is an important factor to aeroponic systems as the amount of oxygen available to the root system depends on it. Still, Gopinath et al. (2017) emphasized that aeroponic systems with larger pumps require greater energy requirements compared with other hydroponic systems.

#### 2.1.3 Aquaponics

**Figure 4:**
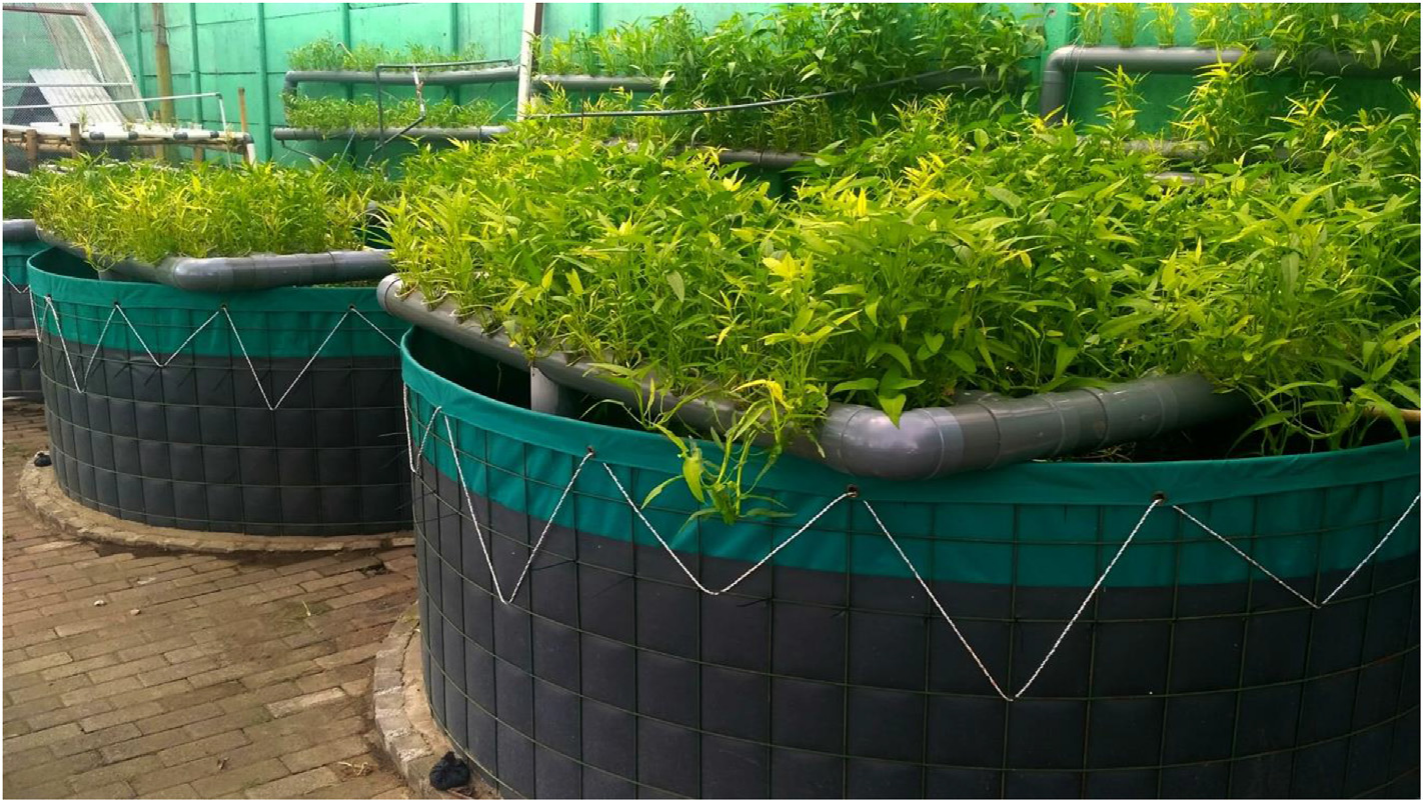
An aquaponic farm where vegetables and fish are grown for food. Photo by HarJac20/Shutterstock.com.

Another form of vertical farming is aquaponics which combines aquaculture and hydroponic systems (Figure 4). The main advantage of this system is the integration of the fish and crop farming, which creates the exchange of nutrients through the water that is shared between the two. It has similar advantages to the hydroponic and aeroponic systems in its efficient use of water, soil-less cultivation, but in addition, it allows plants and fish to grow simultaneously without increasing water consumption (Gooley and Gavine, 2003 in Lennard and Goddek, 2019). When it comes to energy requirements, aquaponic systems are likely dependent on system configuration (e.g., design, species, scale, and technologies) and geographic location (Goddek et al., 2015). A combination study of Life Cycle Assessment (LCA) and Life Cycle Costing (LCC) in Belgium found that energy consumption, infrastructure, and water consumption are the main critical issues in an aquaponic system (Forchino et al., 2018). Furthermore, the main economic burden was associated with the energy consumption, which was responsible for about half of the whole production cost. Therefore, designing a system with a less energy and water demand component is needed towards economic and environmental sustainability.

#### 2.1.4 Digeponics

While aquaponics combines the aquaculture and hydroponics systems, the term “digeponics” is coined by replacing the aquaculture with anaerobic digester in a similar system. More specifically, anaerobic digestion is a process by which organic matter is broken down by anaerobic microorganisms to produce biogas and by-product digestate (Marquez et al., 2020). Digestate is composed of solid and liquid fractions which contains nutrients and can be used as bio-fertilizer. Ehmann et al. (2018) reported 0.58 % and 0.38 % of total nitrogen, 0.26 % and 0.24 % of NH_4_^+^-N, 0.22 % and 0.07 % of phosphorus, 0.46 % and 0.41 % of potassium, and 0.47 % and 0.16 % of calcium contents in fresh solid digestate and liquid digestate fractions, respectively.

One remarkable application is the ‘Food to waste to food’ project which was claimed to be the first efficient method for the utilization of digestate as a growing medium and bio-fertilizer in greenhouses (Stoknes et al., 2016). This project integrated food waste treatment through biogas production, while using the digestate as bio-fertilizer to grow crops, and a new closed dynamic bubble-insulated greenhouse technology where biogas is burned for temperature control. A small-scale bubble-insulated greenhouse was constructed in Norway as a prototype. A heat loss of 0.9 W/m^2^ K (watt per meter squared per kelvin) was measured in a bubble-insulated greenhouse, compared to typical conventional greenhouses which have a heat loss of about W/m^2^ K. This makes the energy demand for the small-scale greenhouse lower of only 10–20 % of the energy consumed (usually derived from fossil fuels) by conventional Nordic greenhouses. Also, the incorporation of anaerobic digestion is advantageous in upcycling the organic agricultural wastes such as the roots and stems of crops, which are regularly produced after each harvest in the farm. However, further studies are needed to successfully up-scale the system and optimize growing conditions of crops in terms of substrate microbiology.

On the other hand, seamless and compact biogas digester design which can be operated in urban setting while not compromising energy production is already under development (*unpublished*). Upon commercialization, anaerobic digestion system can easily be integrated to UA, providing better efficiency to any types of farming system. The same compact system platform can also provide wastewater treatment function to remove excess fertilizer before a necessary water disposal.

### 2.2 Plant biotechnology

Urban community farms also face climatic challenges such as extreme heat and cold. Moreover, crops grown in urban farms can also be threatened by pests and diseases. Aside from factors that can affect the growth of plants, some of the other challenges faced by urban farms include limited space, high labor costs, and high operation costs. While open community farms are subject to environmental factors, vertical farms including indoor farms and greenhouses are operated with full control of conditions such as temperature, humidity, light, water, and nutrient input. The major challenges in such farms are limited space and high operational costs.

How can plant biotechnology address such challenges faced by urban agriculture? Plant biotechnology has paved the way for the development of disease-resistant and climate-ready crops to address the current environmental changes faced by farmers in growing their crops. Biotech plants can be developed by marker-assisted selection (MAS), genetic modification (GM) or genome editing (GE). In MAS, conventional breeding can be made faster by using DNA markers to select for hybrids instead of using phenotypic selection which usually requires longer periods of time. On the other hand, GM involves the use of recombinant DNA technology to change the genetic makeup of organisms. Recombination is the insertion of DNA molecules from different distinct species to produce an improved version of the organism. Finally, GE is the most recent technology that has shown immense potential for application in plant biotechnology. Genome editing is based on the precise identification of short DNA sequences and their deletion, then insertion of new DNA sequence to correct errors or to change the genetic information.

Using plant biotechnology tools, it is now possible to develop crops with desired traits such as resistance to pests and diseases, tolerance to drought, heat, cold or salinity, improved flavor, rapid cycling as well as other superior growth traits. For urban agriculture, the limited space for cultivating crops can be addressed by developing plants with compact architecture and rapid life cycle (O'Sullivan et al., 2020). Using the GE tool *CRISPR-Cas9*, Kwon et al. (2020) targeted the genes responsible for stem length and flowering in tomatoes to create a smaller plant size that can produce fruits in a shorter time span. Dwarfism is a trait that naturally exists in some varieties of crops and has been used to improve other commercial crops. The gene responsible for dwarfism has been identified and characterized in many plant species and used in plant breeding for decades now. Similarly, the genes that are involved in the regulation of flowering time have been extensively studied and shown useful in crop improvement. Targeting these traits, Kwon et al. (2020) created compact varieties of cherry tomato and ground cherry that have the same productivity as the wild-type varieties. This strategy can be applied to other vegetable and fruit crops that can be cultivated either in indoor or outdoor community urban farms. Maintaining a high flowering/fruiting rate for agricultural crops in urban farms can compensate for the high operation costs and will not put the burden on the consumers. Table 1 shows some plants which have undergone genetic modification that may be suitable for UA. Further, Lobato-Gómez et al. (2021) compiled a list of genome-edited fruit-bearing crops of which can be explored for their suitability in UA application.

**Table 1:** Genetically modified plants suitable for urban farming.

Plants	Tools	Characteristics	References
Tomato	CRISPR-Cas9, ​silencing ​three genes: ​*SP5G*, ​*SP*, and ​*SlER*	• Shortened internodes to increase compactness while maintaining ​productivity ​of tomato	Kwon et al. (2020)
Kiwifruit	CRISPR-Cas9-mediated mutagenesis of ​*CEN*-like candidate genes	• Shortening life cycle within a year ​with rapid terminal flower and fruit development	Varkonyi-Gasic et al. (2019)
Lettuce	*Agrobacterium tumefaciens*-mediated ​down-regulation of ​*XTH* ​genes through antisense inhibition	• Improvement of shelf-life of ​harvested leaves	Wagstaff et al. (2010)
	CRISPR-Cas9-mediated ​knocking-out of ​*LsNCED4*	• Increased ​seed germination temperature to 37 °C	Bertier et al. (2018)

While crops that are developed by conventional breeding are more easily accepted by the public, those that involve GMs are still not accepted by the Japanese consumers even though the Japanese government has approved the commercial cultivation of GM crops. The same is true for genome edited agricultural products. However, it is interesting to note that Japan remains one of the top importers of food and feed products developed using genetic engineering from the US. According to USDA Foreign Agricultural Service report, Japan imports 100 % of its corn supply and 94 % of soybean supply, which are mostly GM (Sato, 2020). Genome edited crops are still being evaluated for commercial cultivation in many countries while regulations in different countries are still being established. In 2021, the first GE crop was successfully launched to the Japanese market after Ezura and co-workers developed a GABA-enhanced tomato, making it the world's first GE crop to be commercialized (Ezura, 2022). GABA or γ-aminobutyric acid is an amino acid with human health benefits, particularly useful in the prevention of hypertension. Since GE crops does not contain a “foreign gene” (i.e., transgene-free), consumers might have less bias against them. Once the appropriate regulatory framework for the commercialization of GE crops is established, they could eventually be accepted by the consumers (Ishii and Araki, 2016). Nevertheless, these GM and GE crops have enormous potential in maximizing the productivity of urban agriculture in Japan and other countries.

### 2.3 How can UA help?

Despite the necessity of integrating UA into sustainable city planning, it is only relatively recently that the topic began to gain attention. With the rapid urbanization, the concept of sustainable cities that *“emphasise a balance among economic development, environmental protection, and equity in income, employment, shelter, basic services, social infrastructure and transportation”* became prominent (Hiremath et al., 2013 in Azunre et al., 2019). Although somewhat included in the policy planning, UA was generally moved to the periphery of the discourse with the policies focusing on other aspects of urban development. In particular, the governments in the global south are reluctant to allocate land for UA integration. Therefore, most of the relatively big urban farms are located on the peripheries of the cities due to lower land prices (Azunre et al., 2019).

However, the situation is gradually changing with the realization that UA has profound implications for the sustainability of cities in terms of its economic, environmental, and social contribution. Expansion of green zones in the cities improves air quality, and partial reliance on urban agriculture decreases emissions of greenhouse gases. UA also contributes to local trade development, creating full time employment and additional sources of income. For instance, in Ghana, urban farmers produce most of the exotic vegetables for the region, such as lettuce and spring onions, and supply them to urban markets (Azunre et al., 2019). Furthermore, UA has the potential of becoming a source of sustenance for urban communities and providing impoverished population with necessary nutrition. It gives people access to fresh and chemical-free products while reducing their food expenditures. In developing regions, the percentage of poor households engaging in UA substantially exceeds average-income households (Zezza and Tasciotti, 2010). However, it is not to imply that UA alone can fully sustain urban population, instead, a balance between urban and rural agriculture should be reached to secure cities’ food supply through sustainable practices.

The ongoing COVID-19 pandemic, which disrupted numerous distribution channels and food production processes all over the world, highlighted the urgency of the food security issue. Although no significant fluctuation of the food prices on the global level was recorded during the pandemic, inflation of food prices was present, with low- and middle-income countries sustaining majority of the damage (World Bank, 2021). Population in the developing countries spend a larger portion of their income on food compared to the high-income countries, which puts an additional strain on the vulnerable groups (World Bank, 2021). Restriction on the movements of people and goods further inhibited the access to food on urban markets, thus creating food deficits and causing inflation (FAO, 2020). Unemployment is also on the rise during the pandemic due to the production processes being put on hold in attempts to stop the spread of the disease.

With the combined impact of the reduced income and higher food prices, many households were forced to reduce their expenditure on food and lower their quality standards as a sustenance measure (World Bank, 2021). According to the World Bank (2021), by the end of 2020, approximately 130 million people will face acute food insecurity. Prior to the pandemic, such drastic global-scale reduction in life quality due to food insecurity problems was hardly imaginable. However, the current global food crisis and its repercussions fully demonstrated the urgency of the problem. Hence, the next section will examine how UA can increase its role in playing its part in solving these challenges.

## 3 Different contributions of urban agriculture to city

### 3.1 Economic perspective

UA can be defined as a variety of livelihood systems such as subsistence production and processing which can be adapted to urban situations from the household level to a more commercialized sector (van Veenhuizen and Danso, 2007). From subsistence-oriented motives to large scale commercial production facilities, UA has many different roles for communities in the cities and urban areas. While UA economic benefits are marginal at the community level, it has the potential to contribute to building the resilience of urban communities, especially in coping with economic challenges.

In measuring the economic viability of UA, its economic impacts and profitability are distinguished in three levels: (a) household level, (b) city level, and (c) macro level (van Veenhuizen and Danso, 2007).

At the household level, economic benefits and costs involved in agricultural production such as self-employment, exchange of products, income from sales, savings on food and health expenditures are directly incurred by the urban households. A study in four West African capitals showed that rainfed crops such as maize and cassava are mainly produced for household consumption, while short-cycle and long-cycle crops such as lettuce, cabbage, carrots, and onions can generate monthly income from sales (van Veenhuizen and Danso, 2007). Furthermore, in Ghana, income from irrigated urban vegetable farming was found to be two to three times higher than the average income earned from rural farming (Danso et al., 2002).

At the city level, there are: (a) direct benefits and costs which are not carried by the farmers, and (b) indirect benefits and costs which are in the form of positive and negative externalities. These externalities include the social, health, and environmental impacts of UA in the urban setting. However, comparing different city situations remains a challenge as these impacts depend on policies and legislation existing in the city. One common approach for economists to examine or quantify these impacts is by using cost-benefit framework (Nugent, 1999) although such method should be applied more extensively in analyzing UA's impacts.

At the macro level, effects of UA are felt through its contribution to the national's gross domestic product (GDP) and to the efficiency of the national food system. Moreover, UA products can supplement rural agriculture's limited supply, substitute for food imports, and boost export production of agricultural commodities (Mougeot, 2000). In Kenya, UA has generated the highest self-employment to small-scale enterprises and the third highest earnings overall (House et al., 1993). Unfortunately, studies on economic impacts of UA in the macro level are limited since most research are focused on the household level.

The term ‘economic viability of UA’ can also be ambiguous. Copious literature has discussed the economic viability of either micro-farms, rooftop gardens, greenhouses, or vertical farms to examine the cost and gains of these specific types of UA (Whittinghill and Rowe, 2012; Thomaier et al., 2014; Sanyé-Mengual et al., 2015; Chang and Morel, 2018). These authors realized that different types of UA can have significant variations in economic viability, but they usually take part for the whole and generalize the economic viability of UA based on their specified discoveries. To better understand the difference, some literature of UA's economic viability using different approaches has been summarized in Table 2. It indicates that UA's economic viability is apparent, albeit several economic factors (e.g., proximity, investment and operation costs, capital, and consumer knowledge) should be taken into consideration to assess the benefits and costs in engaging into UA.

**Table 2:** The economic potential of different types of urban agriculture.

Location	UA concerns	Result(s)	References
Ruhr metropolis, Germany	Professional urban and peri-urban farms	• It is less likely to achieve success in densely populated municipalities where various adjustment strategies (e.g., provision of tourism services, using short distribution channels) are not implemented • Farm success is mainly dependent on the farm location thereby minimizing transport costs and offering convenience to the customers • Full-time farmers who are using appropriate adjustment strategies for farm development are more likely to achieve farm success in the long run	Sroka et al. (2019)
Galati, Romania	Integrated aquaponics system: Deep water culture (DWC) and Light expanded clay aggregate (LECA)	• LECA substrate aquaponic technique requires higher investment costs but generates higher income than the DWC technique • Electricity costs represent more than half of the total variable costs value, thus creating a great demand for renewable energy source alternatives	Petrea et al. (2016)
Sicily, Italy	Pilot aquatic plant producing lettuce and Nile tilapia	• Aquaponic farming yields positive operating income and benefit/cost ratio for the first year of experimental activity • Economic viability might be slightly more sensitive to revenue than its operational cost	Asciuto et al. (2019)
Arizona, US	Consumer behavior towards urban farming	• Consumers having subjective knowledge of UA and a favorable attitude towards urban farming increases the likelihood to purchase ​produce from urban farms and grow their own produce at urban farms	Grebitus et al. (2017)
European cities	Economic performance and self-sufficiency of urban gardening	• Most urban gardeners were not motivated ​mainly by ​profit, but of other factors such as safe and healthy food production, source of relaxation, environmental impact, and as a means of socializing • Albeit profit being of second importance, economic productivity of urban gardens can be compared to market production by a substantial amount	Glavan et al. (2018)
Bangkok, Thailand	Peri-urban farming systems (fish, shrimp, rice, and fruits)	• Despite having the highest costs among the four systems, shrimp farms remain to be the most profitable, yielding the highest income, net income per family worker, investment, and input costs	Vagneron (2007)

### 3.2 Social perspective

Regarding the implementation of UA systems within developed countries it is important to acknowledge that integration is taking place within pre-established socioeconomic structures, and not the other way around. In the Global North, the physical and cultural environments encountered by the UA narrative are often distinguished in part by deeply rooted societal structures and potential injustices requiring attention. To this end, systems of inequality can distort the “sustainable” and “social justice” front commonly adopted by UA initiatives, by engulfing operations within socially detrimental processes like eco gentrification (McClintock, 2016). In other words, the new entity is forced to work around pre-existing frameworks, a transition that is often facilitated by policies (Siegner et al., 2018).

Food insecurity and gentrification in cities highlight many of the challenges targeted by urban farming, yet point to external social issues which necessitate attention if UA is to become truly economically viable. Specifically, food insecurity is a manifestation of wider, and deeply embedded inequities, to the extent that expanding agricultural systems into cities does not automatically guarantee improved food security for the residing population (Horst et al., 2017). This is because low-income communities are likely already subject to underinvestment and discriminatory patterns. Farms are thus left vulnerable to falling into “*a corporate food system model of profit maximization and resource use efficiency, subscribing to capitalist logics rather than alternative, social-justice-oriented practices*” (Siegner et al., 2018). These problems are exacerbated when met with the high cost of development pressures, rendering urban produce either unattainable or unaffordable for many. Thus, dialogue surrounding urban farms and inherent potentials becomes unproductive when it is conflated with generalized notions of increased access (Siegner et al., 2018).

Several studies have shown a concentration of urban or community farms in places where they are not most needed to improve food security. That is, organizations have not been strategically distributed throughout the cities in question to the advantage of those who need it most (Horst et al., 2017). There exists a contradiction between utilizing UA to combat food insecurity, and a preconceived notion which employs “greening” as a tool in gentrification to make neighborhoods more attractive to the upper class. That is, the development and presence of green spaces is often followed by increasing property values (Daftary-Steel et al., 2015). In San Francisco, community garden initiatives started by minority groups have grown in recent years, onsetting neighborhood remodeling processes in response to the “beautification” brought about by green spaces (Marche, 2015). Therefore, if UA is to become economically viable by improving upon societal inequities, its implementation needs to be structured to resist gentrification, not contribute to it.

In terms of external social conditions, the most optimal solutions involve attacking systemic inequalities at the core, still policy mechanisms and strategies exist which can help prevent UA integration from succumbing to harmful capitalist tendencies. McClintock (2014) observed the effects of neoliberal policies which served more radical variants of agricultural entrepreneurialism that “*return the means of production to urban residents.*” Regardless of top-down versus bottom-up distinctions, endeavors reflecting a degree of municipal liberalism in practice display the capacity to meet residential needs because of a continued engagement with civic activism (Marche, 2015). This is indicative of a boundary wherein policy capabilities meet the need for civic participation in order to optimize the benefits offered by UA within a society which manifest themselves on a couple of fronts.

The intersection with social injustices is inevitable in the integration process of UA, therefore it becomes beneficial for local governments to include the voices of residents. Given the pernicious tendency to favor “beautified” variations of community farms, the deliberate inclusion of the community in the decision-making process helps to ensure a service-based system geared towards the society. In accordance with approaches posited by neoliberal policies, the secession of regulations thereby clears a space for local voices, enabling structure that is self-sustaining and less susceptible to gentrification (Marche, 2015). Municipal working groups offer a potential solution by filling gaps in formal policy, while departments or focus groups can be organized to meet specialized needs (Deelstra and Girardet, 2000). Food policy councils in Portland and Vancouver for instance are composed of local activists that advise municipal governments in navigating related issues, and draft proposals for project development (Mendes et al., 2008). Meanwhile, councils in New York have held policy makers accountable, providing communities with an extra layer of protection from extensive development or becoming exclusionary (Cohen, 2016). Subsequently, what emerges are channels that propagate mutual relations between public officials and civil society. Co-dependency between the two entities is thus reliant upon active civil participation without absolving government responsibility.

Although UA in isolation is not a viable solution, producers can be situated to work against social injustices rather than being absorbed to uphold an already corrupt system. Attuning control and responsibility of government officials helps make space for grassroot efforts and sufficient interaction with relevant social justice movements taking place in the community. With the support of local councils, policy approaches would benefit by recognizing the intersections and resultant variables within the agricultural sector which allow UA to encompass more than food production and security. Further, utilizing policies in such a manner to extract commonly, or uncommonly, theorized benefits of UA will enable the future economic viability of these projects. However, this is predicated not only upon an inward-looking understanding of the sector itself but a comprehensive perception of the surrounding society to make the most of UA's characteristics in each respective case.

### 3.3 Disaster risk reduction perspective

Here it is worth briefly mentioning the specific functions of UA in the context of emergency crises and post disaster reconstruction. The impacts of disasters on urban areas have been exacerbated by the effects of global warming. Effects are particularly acute in developing countries, water-stressed countries, as well as coastal and low-lying regions. Many cities are also predisposed to the risk of food supply chain disruptions, which in turn often disproportionately affects the urban poor, elderly and the disabled (Dubbeling et al., 2019). Furthermore, rapid urbanization and mass migration into city centers in developing countries can often lead to competing demands, diminishing resources, and overextended infrastructure systems. On these points, UA offers several potential benefits to help mitigate the negative impacts incurred by disasters, expedite post-disaster reconstruction processes, and contribute to overall urban and livelihood resilience.

As mentioned, one of the primary impacts of disasters on urban areas relates to supply chain disruptions. Dependence on imported food often leaves even very developed cities vulnerable to sudden food depletion. The severity of import-dependence in many cities is exemplified by the fact that cities such as London are never more than five days away from food depletion (Adam-Bradford, 2010). Meanwhile, economic crises can result in rising food prices compounded by unstable incomes, which can push the urban poor further into poverty (Adam-Bradford, 2010). Thus, following a crisis, urban populations may resort to informal markets to sustain their livelihoods, this includes UA.

The existence of local agricultural food production helps to reduce vulnerability to supply chain disruptions in times of crisis. For example, urban areas in developed countries have experienced first-hand the impacts of food supply shortages during the recent COVID-19 pandemic. In Tokyo, the existence of UA has helped to mitigate some of these negative effects by shortening the supply chain and providing residents with direct access to local produce (Yoshida and Yagi, 2021). In several cases, UA farms have been able to increase sales since the start of the pandemic due to the country's stay-home campaign and increasing consumer demands for local marketing channels (Yoshida and Yagi, 2021). These short supply chains or direct marketing schemes employed by Tokyo's urban farms thus represent a specific resilient attribute of UA that has supported food security in a time of crisis.

In addition to enhancing food security, much of the literature has emphasized the role of UA as a livelihood strategy. Specifically, that its contributions during disaster risk reduction and management extend beyond addressing the immediate challenges of food insecurity. For example, during economic crises, UA can help to subvert income insecurity and marginalization by stimulating ‘green job’ creation and diversifying income sources for many households. This helps to alleviate some of the immediate pressures faced by the urban poor by expanding their coping capacity in times of financial distress (Dubbeling et al., 2019).

Besides economic benefits, UA also presents numerous social benefits that should not be overlooked in the context of risk reduction and urban resilience. The experiences of refugee camps offer a constructive illustration of UA's social dimensions. A study conducted by Tomkins et al. (2019) traced the role of UA in Iraqi refugee camps as many have evolved into ‘accidental cities’ since the start of the Syrian Civil War in 2011. Of the camps surveyed, refugees generally had adequate access to food supplies due to the prominence of humanitarian aid. Therefore, instead of relying on UA for sustenance purposes, gardens were often associated with benefits such as promoting social cohesion and providing a healing space from trauma. These multifaceted benefits are further exemplified by the 16 different types of gardens identified in the study, which range from street gardens to ornamental planting practices (Tomkins et al., 2019).

Given the wide-ranging functions of UA in disaster risk reduction practices, its implementation should be situated within a more comprehensive risk reduction strategy. In other words, UA should be integrated with wider development objectives if municipalities are to make the most of all it has to offer. For example, in the case of the refugee camps, the existence of UA has stimulated development of other constructive infrastructure thereby bolstering sustainable practices within camps. This has included Sustainable Drainage Systems, which have facilitated water mobility, improved water quality, greywater management and reduced pollution and erosion (Tomkins et al., 2019). In other cities such as Beijing and Toronto, UA has been incorporated into municipal climate change action plans, while its economic benefits have supported “slum-upgrading” programs in many South American countries (Dubbeling et al., 2019). In particular, arid climates such as in Burkina Faso, UA has been implemented as a part of efforts to lower surface temperatures and reduce impacts of the urban heat island effect (Dubbeling et al., 2019). It can therefore be seen how the efficient integration of UA can help municipalities meet multiple development objectives simultaneously. However, it is important to note that many such benefits are predicated on government involvement and effective coordination between municipal authorities and local civil society groups.

Oftentimes, the realization of UA's full potential has been inhibited by a lack of governmental recognition and technical assistance. This is especially true in post-disaster contexts, wherein agricultural production is easily overlooked in times of crisis. It is not uncommon for relief operations to leave recovering communities dependent on external food aid. It is for these reasons that agriculture-related activities should be implemented during early stages of the post-disaster cycle (Adam-Bradford, 2010). The fragility of UA systems has more recently been highlighted by the impacts of COVID-19. One study conducted on urban and peri-urban farms in São Paulo found that a lack of municipal support exacerbated pre-existing shortcomings. Namely, a lack of technical assistance, an inability to diversify commercialization channels, and difficulty accessing inputs. Furthermore, noncommercial community gardens were unable to significantly contribute to food security due to restrictions and lack of formal recognition by the government (Biazoti et al., 2021). Thus, if UA is to advance disaster risk reduction, it will require more direct engagement with government authorities to promote integration with long term development goals.

### 3.4 Health and well-being perspective

UA can alleviate poverty and food insecurity, while also improving the health of city residents and preserving the environment (Foodtank, 2017). In addition, urban green space is a necessary component for delivering healthy, sustainable and livable cities for all population groups, particularly among lower socioeconomic groups (WHO, 2017). Because of the continuing shift of population to urbanized areas, studies on how urban nature can be utilized as a tool to reduce health risks have been increasing but with varying results.

Most urban areas, like for example New York City, lack vacant land for green space, making rooftops an important space for greening. In such a case, UA has great potential to help mitigate the city's public health problems on obesity and diabetes which are correlated to inadequate access to fresh, healthy food retail (Ackerman et al., 2014). Fruits and vegetables are the most common types of food that can be cultivated on a rooftop greening. Through the increase in fruit and vegetable cultivation and consumption, improving health conditions, and reducing poverty may be achieved (Orsini et al., 2013). In Tokyo, other than as a source of fresh and safe products, UA serves as a resource for recreation and well-being, including a space for personal leisure and spiritual comfort (Moreno-Peñaranda, 2011).

Studies on the association between green spaces and general health, and the mediators of this association have been reported as well. Dadvand et al. (2016) investigated whether the presence of green space can attenuate negative health impacts of stressful life events using a quantitative data of a representative sample of Dutch residents. The results showed that only the relationships of stressful life events with the number of health complaints and perceived general health were significantly reduced by the amount of green space in a 3-km radius. However, buffering effects of green space were less pronounced for mental health than for physical and general health indicators and provided a conservative and rather limited test of the buffering effects of green space that is close to home. Another study assessed the association between greenness exposure and subjective general health (SGH) through evaluation of their mediators such as mental health status, social support, and physical activity (van den Berg et al., 2010). Using the data obtained from a population-based randomized sample of adults residing in Barcelona, Spain, the study revealed mental health status, perceived social support, and to less extent, physical activity, to be more impacted by residential surrounding greenness than subjective proximity to green spaces (van den Berg et al., 2010). Further, a study among youth living in the city of Plovdiv, Bulgaria was conducted to compare single and parallel mediation models— estimate the independent contributions of different paths— with several models that posit serial mediation components in the pathway from green space to mental health (Dzhambov et al., 2018). The researchers found that higher restorative quality in the neighborhood brought by higher perceived green spaces was directly associated with better mental health and promoted more physical activity and more social cohesion, and in turn, indirectly led to better mental health. Hence, direct and indirect positive effects of green spaces, and in extension UA, on the health and well-being of urban dwellers should incentivize UA's integration in urban planning because their long-term impact on the population's economic productivity and healthcare cost can bring the city's finances into better position when compared with short-term gain from allowing maximization of urban space for commercial use.

### 3.5 Environmental perspective

Environmental risks often emerge as agricultural practices shift into city centers. Such risks may pertain to the production of goods and services by farms, or they may appear as negative externalities in the surrounding community. For instance, increased levels of pollution in cities can diminish the quality of urban-grown products, generating health risks for consumers (Tuijl et al., 2018). Meanwhile, the use of certain pesticides, herbicides, and fertilizers in the process of farming can generate additional risks for residents and damage local biodiversity. Under such circumstances, farming practices may become environmentally detrimental, or unwanted in heavily populated regions, particularly of those commercial urban farms (McDougall et al., 2019). While these additional risks are minimal for small-scale UA, the practice of commercial-scale UA using soil-base farming will bring the same risks as agro-industrial farms do on their surrounding environment. Buscaroli et al. (2021) identified three cases where plant protection products (PPP) used in UA may cause harm to its environment, these are, “*1) disregard for precautionary limitations, 2) misuse of authorized active substances, and c) use of unauthorized substances.*” While these are preventable, the lack of supervision and regulation on backyard UA may suggest that the risks are still present albeit minimal. To avoid risks, it is highly recommended to regulate the type and size of farming in cities. For example, mandating the use of vertical farm technique when establishing a commercial-scale UA will prevent these PPP risks in urban communities while bringing commercial-scale source of food within cities.

The relationship between the agricultural sector and the environment is defined in two senses by the latter. Namely, the environmental impact induced through alterations made by farming practices, and subsequently the kind of environment that is produced by incorporating food production in the given region. This is true of urban and rural systems alike, illuminating the push to reconcile modern agrarian methodologies with environmental conscious regulations (Kalen, 2011). Regarding environmental risks, though many of the associated negative externalities are well researched, and a degree of precedence exists in this policy sphere, exemptions have often been made in agriculture, generating harmful regulatory gaps (Schneider, 2010). It is therefore important that farming methodologies being brought into city centers act in harmony with wider environmental policies and standards rather than go unregulated. Such policies can be deemed as an effective solution to help correct negative externalities and risks placed on the environment.

One of the most prominent environmental risks faced by UA in contemporary societies has been the navigation and risk management associated with environmental contamination. Specifically, the anthropogenic pollution of soil and air as a result of industrial activities, transportation, mining, sewage, and fossil fuel combustion. The ultimate impact of environmental contamination of produce is dependent on several factors such as the quantity and type of pollutant present, how long the produce remains in the soil, and similarly the kind of crop being exposed. Vegetables like lettuce and cabbage risk greater exposure to atmospheric particles on account of the greater surface area of leaves, while root vegetables are more vulnerable to soil contaminants. Duration of growth will also increase or reduce the amount of exposure to any pollution present, and so herbs like thyme, which are grown year-round, become more susceptible to absorption (Aubry and Manouchehri, 2019).

Regarding contamination, lead is a commonly cited concern for urban farmers utilizing soil-based methods of crop cultivation. Leaded-gasoline and paints were widespread several decades ago, despite the phase-out of such products many urban sites today continue to test positive for varying levels of contamination (LaCroix, 2014). However, aside from low-growing and root vegetables, the lead uptake of plants is generally low, and risks of bioaccumulation remain small (Brown et al., 2016). One study concluded it to be highly unlikely that human consumption of food grown in lead-contaminated soils would result in elevated blood levels of the component. Additionally, that elevated levels present within the soil pose minor risk to UA in general (Brown et al., 2016).

Still other forms of urban air and soil pollution do exist that could impede more seriously upon the uptake of UA systems in certain cities. For instance, old industrial sites may be more prone to different forms of contamination depending on the type of activities once conducted on the land (LaCroix, 2014). While produce grown near roads may risk contamination from vehicles. One study in Italy found a higher uptake of elements such as, Ba, Cu, Pb, Sb, Sn, V, Zn in vegetables grown within close proximity to roads (Antisari et al., 2015). Simultaneously, a high soil pH has also been documented to accelerate plant uptake of contaminants found in the earth, especially the bioavailability and toxicity of Pb and Cd (Chang et al., 2014). Finally, though the presence of these pollutants may pose health risks by way of vegetable consumption, another significant pathway for exposure is through the direct ingestion of soil and dust particles (Paltseva et al., 2020).

However, these risks may be subverted depending on the type of UA that is being utilized. For instance, farming technologies associated with indoor farming, hydroponics, aquaponics, may help to minimize the impacts of soil and/or air pollutants generated by human activity. Though the use of alternative farming mechanisms can help mitigate risks posed by urban pollution, its employment is succeeded by other changes in production that can affect the overall economic viability and sustainability of UA. A simple example of this might be how the use of indoor farming shields crops from air and soil pollution in cities, but may simultaneously require greater energy consumption for climate control systems (Aubry and Manouchehri, 2019).

From the opposite perspective, agricultural practices which are focused in producing high-quality products, especially those which are utilizing terroir approach, will be more inclined to improve the environment and local ecosystem condition where the UA are located. Using terroir concept, the interaction between local environment and ecosystem characteristics as well as the local agriculture knowledge and practices can directly influence the characteristics of agricultural products (Ashardiono, 2019). In the premise that these high-quality products command better profit, UA which utilize terroir approach will have more incentive in demanding urban policies which promote better environmental condition around their site. As the following example illustrates, UA production tradeoffs can be overcome through policies, thereby heightening long term viability.

## 4 Policies in urban agriculture

To accommodate the multiple functions of UA, in addition to the sector's intrinsic diversity, respective urban policies require a degree of structural robustness in ensuring proper integration. By and large however, policies remain limited in scope, and incapable of sufficiently implementing systems within respective municipalities (Orsini, 2020). The more recent emergence of UA initiatives helps to explain some of these policy gaps and lack of formal recognition. Respectively, since the adoption of the Support Group on Urban Agriculture (SGUA) in 1992 by the UNDP's Urban Agriculture Advisory Committee (UAAC), developed states have begun to gradually incorporate policy support for UA into national legal frameworks (van Veenhuizen and Danso, 2007). Take for instance, the lack of specific provisions for city farms in the EU's rural development policy between the years 2007 and 2013 (McEldowney, 2017). Similarly, in the United States, formal recognition of urban food production in the context of planning only took hold in 2007 with the establishment of the Policy Guide on Community and Regional Food Planning (American Planning Association, 2007). Moreover, despite the growing popularity of community gardens, farmer's markets, and urban farms in Australia, the country had yet to implement similar strategies or policy mechanisms as of 2019 (Sarker et al., 2019).

Siegner et al. (2018) contrasted supposed implementations with observed realities as a product of shortcomings within urban planning political frameworks. Theoretical work in cities like Cleveland has shown the production capacity of urban farms to meet local demands almost entirely on the assumption of robust policy and planning support. This observed disparity between theory and practice, is underpinned by issues of inequality that have yet to be directly addressed (Horst et al., 2017). Once again, much of this can, and has been attributed to the nascent industry and developing foundation of related academic literature. Fully fledged legislative systems, extending beyond surface level benefits of UA and into issues of economic inequities, therefore need to be established on the grounds of empirical analysis to improve functions of future adaptations (Stewart et al., 2013).

Whilst evolution in urban planning has taken place during the 21st century, development has remained within boundaries defined by the knowledge and intention of policymakers. A substantial amount of academic literature exists introducing social benefits of UA, and how policy mechanisms may help realize such potential Horst et al. (2017), e.g., outlined food justice goals in the United States and Canada, a characteristic of UA that is often celebrated and looked into by initiatives in developing countries. In other words, it is deployed as a solution to food injustice, or a strategy to minimize economic disparities in urban spaces. Despite this, Horst et al. (2017) noted that “*without explicit valuation of food justice*” policy mechanisms existing congruent to this common, well-researched stance will ultimately fall short of uplifting the disadvantaged communities they seek to target. Additionally, UA is only part of a food justice solution, and that “*there is a distinction between alleviating symptoms of injustice . . . and disrupting social and political structures that underlie them*” (Reynolds, 2015).

To this extent, even with commonly referenced and targeted goals such as food justice, purported benefits of UA are not a given in the absence of robust policy frameworks. The researched socioeconomic benefits of UA extend beyond such mainstream functions, and the sector's rapid development has onset advancements currently not accounted for in policy regimes. Consequently, as the next section seeks to detail, this stifles development of legislation targeting lesser-known features in need of support, such as hygiene or regulatory challenges presented by livestock and digital farming, respectively.

### 4.1 Policy challenges

#### 4.1.1 Livestock rearing as part of urban agriculture

The inclusion of agriculture into populated metropolitan areas has given rise to hygiene concerns particularly around raising livestock. Though less of an issue for plant-based farming, discourses for animal husbandry center predominantly on tradeoffs made between food security as a benefit of UA and maintaining public health standards (Butler, 2012). Thus, there exists a dichotomy whereby overly strict standards can result in a restrictive, exclusionary space, whilst undeveloped ones may promote volatile developments subject to inconsistencies (Butler, 2012). Urban livestock initiatives have engendered a kind of shock to municipal policy systems on account of reintroducing animals into city centers. This is in direct contradiction with the expulsion of farm animals to rural spaces at the height of the industrial revolution specifically for sanitation reasons (Butler, 2012).

The city of Oakland's attempt to amend its home occupation permit provides one such example whereby the products of animal husbandry were overlooked by policy makers. McClintock et al. (2014) observed that in the state of Seattle, residents are not required to obtain a permit to sell produce grown directly from their property. At the state level this law is inclusive of plant and animal farming alike. However, the amendment made by the city of Oakland in 2011 to its related local ordinance on home occupation failed to mention the inclusion of animal products such as eggs and honey from these permit exemptions. So, although state permits were not required, failure of explicit omission on behalf of the local jurisdiction complicated the process for its respective residents seeking to sell such products. Such transitory processes of including animal farming in developing UA policies has highlighted the fact that there remains a dearth of certain regulations and specifications tailored to livestock.

Although municipal codes have evolved substantially, they continue to require reconfiguration to accommodate the possibility of urban livestock. Several variables including species type, real estate, and animal cruelty laws exist on this front to structure codifications. One study conducted on livestock owners in several cities across the United States found considerable diversity in the types of regulations faced by farmers (McClintock et al., 2014). Ordinances between states ranged from area requirements, restrictions on animal numbers, noise, hygiene, to some combination of regulations, or none at all. A vast majority of respondents with chickens were found to be in violation of municipal setback codes, with some making the case that distance from property should be contingent upon other factors such as agreements with neighbors (McClintock et al., 2014). To this end, the argument is made to establish a middle ground wherein policy mechanisms adopt a case-by-case basis while simultaneously leaving room for potential variants that may emerge (McClintock et al., 2014).

#### 4.1.2 Digital farming as new form of urban agriculture

As UA systems have evolved, they have come to intersect with other industries, such as the tech sector, which has enabled the development of new dimensions. This includes elements such as automation, software integration, and silicon-based hardware (Carolan, 2020). Digitized alternatives are being adopted by rural and urban farmers alike as they can help increase output and optimize production. Vertical farming offers several common examples of how technologies have been integrated into the agricultural sector thus far. For instance, the use of HVAC (heating, ventilation, and air conditioning) systems helps to maintain suitable environments for vertical farm crops. In order to do so, systems make use of automated monitoring operations that help track environmental variables like temperature and humidity (Kalantari et al., 2017). Such systems make use of sensors and actuators to build up a database of information about the surrounding environment, eliminating the need for human management (Kalantari et al., 2017). However, as a study conducted by Carolan (2020) on the topic of digital urban agriculture (DUA) exemplifies, these advancements have complicated regulatory efforts so desperately needed.

Notably, farms associated with DUA were found to enjoy greater ease of integration on the policy-front due to blurred definition lines and the absence of laws specifically targeting the emerging sector. Findings from the study suggested that due to the hybrid nature of DUA, farms often do not fall neatly into either agriculture or technology sectors. This presented planning challenges when it comes to zoning laws. Rather than being classified with traditional UA, by taking on the identity of the tech sector, digital farms were almost indiscriminately faced with fewer zoning restrictions. Again, this was because initiatives were perceived as categorically different from UA practices that lacked the “digital” tag at the front (Carolan, 2020).

Subsequently, lax zoning approaches often favored land allocation to digital farms over traditional UA. In doing so, growing numbers of digital farms were more likely to depress local market prices by selling commodities at breakeven prices. Such phenomena threaten other local sellers as “digitized” operations grow and ramp up production in the absence of adequate policies (Carolan, 2020). DUAs are just one instance of UA's rapid expansion into other industries, a characteristic requiring diligence and consideration on behalf of policymakers to combat harmful regulatory grey areas. To this end, achieving economic viability hinges upon the decision-making process to create an environment that is not only conducive, but responsive to these types of changes.

##### 4.1.2.1 The case of Gotham Greens

Established in 2009, Gotham Greens offers one such example of a “digital” urban farming operation. The organization's flagship greenhouse, situated in Greenpoint Brooklyn, New York, is characterized as a rooftop hydroponic commercial farm. Otherwise referred to as Controlled Environment Agriculture (CEA) the farm utilizes various advanced technologies to help ensure high output efficiency alongside year-round production. This is inclusive of computer systems that manage internal temperatures and irrigation. Moreover, the installation of solar photovoltaics, advanced ventilation systems, and high efficiency pumps and fans further seeks to optimize energy efficiency of the greenhouse (Al-Kodmany, 2018). Since its establishment the organization has opened additional farms at two other locations in New York as well as one in Chicago, expanding production and its consumer base (Reynolds and Darly, 2018).

Construction of the flagship farm at Greenpoint was completed in 2011 following the introduction of new zoning regulations within the state of New York. Specifically, those that enabled Gotham Greens to secure zoning approval eliminated height and bulk restrictions that had previously affected rooftop farms and gardens in the city. Changes in said laws emerged in 2010 in response to increasing awareness for UA initiatives, and particularly sought to encourage and accommodate the development of CEA in urban areas (Meier, 2011).

Policy development in favor of vertical farms is reflective of a trend in the recent decade to support farms associated with high-tech systems like that of Gotham Greens. Accordingly, this resulted in the emergence of other CEA farms around the same time in New York, including Brooklyn Grange, Eaglestreet Rooftop Farm, and Square Roots to name a few (Reynolds and Darly, 2018). The driving force behind policy development, or the relaxation of restrictions specifically pertaining to “digital” operations, has been on the assumption of their sustainability and energy efficiency. However, though Gotham Greens has sought to optimize its energy use through advanced computer systems, some studies have suggested that energy efficiency is not ubiquitous across all CEA initiatives. For instance, a study conducted by Barbosa et al. (2015) found that compared to traditional, soil-based farms, rooftop farms heavily reliant on artificial lighting provided by LEDs were less energy efficient.

In terms of its economic viability, Gotham Greens has been recorded to “produce 7–8 times more food than traditional farming” on account of its technology-dependent efficiencies, and year-round production. Coupled with the fact that the organization was the only supplier of fresh food during Hurricane Sandy, these characteristics appear promising in the context of food security (Al-Kodamy, 2018). However, as Carolan's study highlighted, it was the production surpluses by large commercial “digital” farms like Gotham Greens which can harm smaller agricultural businesses (2020). Furthermore, in observing the growing prominence of rooftop and hydroponic farms, Dimitri et al. (2016) discovered that many displayed a tendency to be profit-oriented and reported higher sales than their more traditional competitors.

Regarding employment, Goodman and Minner (2019) noted that opportunities generated by CEAs overall in New York have proven limited. This is on account of the dominance Gotham Greens currently withholds over the sector, a vast majority of which are in low-paying positions. Even more so, having received an automation grant in 2016, seeking to improve efficiency further, many of these jobs became vulnerable to replacement by machinery. To such an extent, policy development has taken place in New York in support of UA. However, as the example of Green Gotham demonstrated, many of these policies have acted predominantly in favor of initiatives backed by advanced technologies on the assumption that they offer more sustainable and economically efficient alternatives.

The aforementioned instances highlighted a tendency for political frameworks to lack the functionalities that prompt efficient incorporation of agriculture into cities as they overlook the nuances of emerging practices. Here urban planners may benefit in drawing from the related experience of recreational green spaces. Such green spaces have thrived in recent years under comparatively greater social and political support. As Orsini (2020) noted, “*policies exist for the promotion of green spaces in the city for ecological-environmental, aesthetic-recreational, and social-educational purposes.*” One study conducted in the United States found that between the years 2001 and 2007, a total of 204 bills related to park improvement and green space support were passed. The bills covered a wide range of dimensions including, funding, outreach, preservation, recreational activities, and safety. The diversity and quantity of bills passed were thus indicative of “*a continued commitment to improvement and reinvention of existing policies*” in the states represented by the study (Kruger et al., 2010). Should a similar foundation be tailored towards agricultural purposes, UA may become more readily accessible (Orsini, 2020).

Considering the multifaceted potentials of UA integration, the fundamental dimension of policy becomes apparent in addressing the current realities and challenges. Urban land allocation to agriculture can have social, economic, and environmental value-added benefits, necessitating consideration for landscape multifunctionality. These are inclusive of ecological functions like biodiversity protection and nutrient cycling, as well as social cohesion factors such as recreation, health and well-being, and educational opportunities (Artmann and Sartison, 2018). Specific instances exemplifying such multifaceted potentials have been discussed in section 3, which prompted the need of further support in constructing more robust legislative systems to improve initiatives for future adaptations (Krikser et al., 2019).

#### 4.1.3 Educational opportunities

Similar to the environmental protection and development of UA, policymakers also withheld the capacity to promote educational opportunities for urban farmers. In supplying individuals with the necessary knowledge and tools to make the most sustainable decisions, cities can cultivate human capital and ensure maintained success of UA initiatives irrespective of external policy changes (Deelstra and Girardet, 2000). It should be noted that even when left unregulated, farmers have begun reducing pesticide use independently, showing a preference for more organic alternatives (Brown and Jameton, 2000). Community gardens have also opted out of synthetic chemicals in favor of less environmentally damaging methods such as composting and hydroponics (Tendero and Phung, 2019). These more sustainable, eco-friendly alterations are often a product of the intentions that commonly motivate the demographics entering the UA sector.

The values generated by environmental conservation and activism efforts are compatible with those put forth by UA and can therefore influence the behavioral intentions of urban farmers. Educational background, in particular, has a notable impact on the perceived behavioral intentions of farmers (Kopiyawattage et al., 2019). Accordingly, while producers may act on the best of intentions, a lack of knowledge and access to resources can result in mistakes or poor decisions in the context of environmental well-being (McDougall et al., 2019). Given the gravity of educational opportunities, governmental policies can and should situate themselves to promote sufficient pedagogical means for urban producers so that they may more effectively carry out these intentions (Siegner et al., 2018).

In this context, the conduct of UA may be divided into two broad categories, those operated by small or family farms, and commercial size operations. Different operational scales of UA require different skill sets and knowledge. Educational approaches should therefore take into consideration these esoteric distinctions to better equip farmers with information that is relevant to the type of farming at hand. For instance, small-scale farmers may benefit from a detailed understanding of composting practices and cultivation methods to improve overall efficiency and reduce labor costs (McDougall et al., 2019). Similarly, to reduce environmental impacts, improving the carbon literacy of small-scale and community farmers could also improve consumer choices made by these farms (Sharp and Wheeler, 2013).

In particular, some countries and cities seeking to expand UA projects have already started implementing educational and training programs to support local farmers. For instance, the state of California's Cooperative Extension has adopted educational and assistance programs geared towards the support of UA. One such example is the Small Farm Program (SFP) which assists and supports the state's smaller scale urban food producers (Reynolds, 2010). Additionally, California adopted the Urban Agricultural Incentives Zone Act in 2013 which has allowed cities to employ tax incentives for agricultural land-use in designated zones. Significantly, the act encompasses the use of land for educational purposes relating to agriculture (Reynolds and Darly, 2018).

The achievement of high sustainability in urban farms is contingent upon the training and knowledge procured by producers. This contrasts the tendency of recreational farmers to make less sustainable choices, resulting in low efficiency of material and labor inputs (McDougall et al., 2019). This may be addressed by developing education policies and training opportunities for farmers and the community as a whole. Regarding developments within the sector itself, such as new technologies, training programs and workshops aid farmers in updating applied methodologies. Subsequently, the presence of direct farm-to-consumer markets can incentivize farmers by ensuring the profitability of operations. Governments can help ensure that organizations and institutions have the necessary financial means of providing educational opportunities for the surrounding community. Similarly, educating community members helps in creating jobs for low-income households (Carolan, 2020).

Conversely, while local governments can bolster productivity and sustainability of UA, education becomes another benefit of integration as awareness is generated amongst residents concerning topics like nutrition and food production (Tuijl et al., 2018). Promotion of education through agriculture on the policy front thus comes full circle as farmers are equipped with techniques which improve production quality whilst exposure to such practices helps generate more conscious consumers in the community (Horst et al., 2017). Such advantages are demonstrative of alternate societal contributions UA has to offer.

## 5 Conclusion

The economic profitability of UA is highly dependent on its size, type, price competitiveness, and consumers’ perceived value of produce beyond uses as food. Despite its highly relative profitability, UA has many different roles for communities in cities and urban areas, from subsistence-oriented motives to large scale commercial production facilities. Through UA, a household can reduce its expenses by producing its own food, thus leading to savings in their household budgets (Smit and Bailkey, 2006). Furthermore, for a household that produced more than their consumption needs, they can sell the production surpluses and generate additional income for their household. In a more commercially oriented UA, the local community and households will be able to receive income by becoming agricultural laborers in the production facilities or by producing the necessary agricultural inputs such as compost and fertilizer for UA. Additionally, these community and household members can also conduct food processing activities and market food products to gain further income. Among these economic benefits beyond profit, UA can also help provide a healthier diet and nutrition to the urban poor (Zezza and Tasciotti, 2008). Based on these potentials, the level of food security and health conditions of the urban poor communities can be increased through UA activities (Poulsen et al., 2015). For the general urban communities, UA will increase the availability of fresh and affordable foods like vegetables. UA complemented the urban food supplies from the rural agriculture by lessening its dependence on off-seasons food imports, while also act as a buffer when there are reduced supplies, thus flattening the price/variety seasonality (Battersby and Marshak, 2013). Other roles of UA can be embedded as one of the elements in the urban infrastructure, providing several ecosystem services to the urban environment as part of the green and blue infrastructure, whereby maintaining green open spaces and vegetation cover, UA can help improve the urban microclimate, and physical and mental health of urban dwellers. On risk-prone areas such as floodplains, UA can help in stormwater management by controlling the infiltration rate of excess stormwater (Dubbeling and de Zeeuw, 2011). Local food production can reduce GHGs emissions and contribute to a low carbon economy because of shorter supply chains and the amount of fossil fuels used in transportation. Encouraging food production close to cities helps in reducing the ecological footprint of the city, increasing the synergy between urban domestic, industrial sectors, and agriculture (Smeets et al., 2007). With a local food provision, cities will be able to strengthen their resilience (de Zeeuw et al., 2011) and self-reliance in coping with natural disasters and increasing their capacity in adapting to climate change. Local food production will act as a safety net for urban communities during disasters and emergencies when the flow of food distributions from the rural areas failed to reach the urban areas. UA will also reduce the vulnerabilities in urban communities during times of economic hardship (McClintock, 2010), as UA will not only serve as a buffer for food security but also alleviating potential unrest in the communities (Moore, 2006). Therefore, while UA may not be directly profitable, its economic viability is brought by its multidimensional beneficial impacts on the urban environment, social well-being, disaster preparedness, and sustainability.

On the other hand, UA has a potential to be economically profitable as a commercial-scale food producer in a closed system and controlled environment such as vertical farms, plant factories, and greenhouses (Specht et al., 2016). The technologies for this type of UA are already rapidly advancing to increase efficiency and consequently profitability. The integration of digital technology into vertical farms to increase automation, control, and efficiency, incorporation of compatible urban renewable electricity and bio-heating to sustainably power the increasing energy demand of more complex system, and utilization of CRISPR-Cas 9 genetic editing tool to design crops with compact architecture and rapid life cycle to grow in confined space are the current development pushing UA to not only be profitable, but also produce high-quality agricultural products where urban consumers will have assurance on the safety standards of food products.

While the resurgence of UA among cities worldwide has been mainly driven by the public and private sectors, the role of policy makers is an integral part of UA revolution to successfully integrate UA practices in cities. Existing policies and regulations, land prices, availability of urban markets, as well as the prices for agriculture commodities strongly influenced UA activities (de Zeeuw et al., 2011). Its current situation is similar to the early days of renewable energy in the market, particularly solar power. Part of solar power success, aside from the technological and manufacturing advancement, is the monetary incentive policy on both the adopters of technology and their consumers. Hence, government policies which are conducive for UA and properly formulated in the framework of systems approach, can further help increase economic viability of UA while bringing positive impact on food security, social justice, environmental quality, health and well-being, climate change mitigation, and disaster risk reduction.

## Declarations

### Author contribution statement

All authors listed have significantly contributed to the development and the writing of this article.

### Funding statement

This research did not receive any specific grant from funding agencies in the public, commercial, or not-for-profit sectors.

### Data availability statement

Data included in article/supp. material/referenced in article.

### Declaration of interest's statement

The authors declare no conflict of interest.

### Additional information

No additional information is available for this paper.
