# Corrigendum to “A review on urban agriculture: Technology, socio-economy, and policy” [Heliyon 8(11), (2022) Article e11583]

**DOI:** 10.1016/j.heliyon.2023.e13165

**Published:** 2023-01-28

**Authors:** Grace Ning Yuan, Gian Powell B. Marquez, Haoran Deng, Anastasiia Iu, Melisa Fabella, Reginald B. Salonga, Fitrio Ashardiono, Joyce A. Cartagena

**Affiliations:** a College of International Relations, Ritsumeikan University, Kita-ku, Kyoto 603-8577 Japan; b College of Global Liberal Arts, Ritsumeikan University, Ibaraki, Osaka 567-8570 Japan; c Graduate School of Economics, Ritsumeikan University, Kusatsu, Shiga, 525-8577 Japan; d Institute for Advanced Education and Research, Nagoya City University, Mizuho-cho, Mizuho-ku, Nagoya 467-8501 Japan; e College of Policy Science, Ritsumeikan University, Ibaraki, Osaka 567-8570 Japan; f Graduate School of Bioagricultural Sciences, Nagoya University, Furo-cho, Chikusa-ku, Nagoya 464 -8601 Japan

In the original published version of this article, the acknowledgements section omitted some information. This has now been corrected to “G.P. Marquez and R.B. Salonga would like to acknowledge Ritsumeikan University and Nagoya City University, respectively, for their internal research grant support.” The authors apologize for these errors. Both the HTML and PDF versions of the article have been updated to correct the error.

